# Anti-Inflammatory Effects of Heat-Processed *Artemisia capillaris* Thunberg by Regulating I*κ*B*α*/NF-*κ*B Complex and 15-PGDH in Mouse Macrophage Cells

**DOI:** 10.1155/2021/5320314

**Published:** 2021-06-07

**Authors:** Akhtar Ali, Junsik Lim, En Hyung Kim, Jong-Hyun Lee, Shin Seong, Wonnam Kim

**Affiliations:** ^1^Cnh Center for Cancer Research, Gangnam-gu, Seoul 06154, Republic of Korea; ^2^Division of Pharmacology, College of Korean Medicine, Semyung University, Jecheon 27136, Republic of Korea; ^3^Department of Dermatology, Bundang Jesaeng General Hospital, Seongnam, Gyeonggi 13590, Republic of Korea; ^4^Department of Natural Medicine, College of Pharmacy, Dongduk Women's University, Seongbuk-gu, Seoul 02748, Republic of Korea; ^5^Soram Korean Medicine Hospital, Gangnam-gu, Seoul 06154, Republic of Korea

## Abstract

Growing evidence suggests that dietary nutrients in herbs and plants are beneficial in improving inflammatory disorders. *Artemisia capillaris* Thunberg (AC) is a traditional herbal medicine widely used in East Asia to treat pain, hepatotoxicity, and inflammatory disorders. Heat processing is a unique pharmaceutical method used in traditional herbal medicine to enhance the pharmacological effects and safety of medicinal plants. This study demonstrates the anti-inflammatory effects of heat-processed AC (HPAC) in lipopolysaccharide- (LPS-) treated mouse macrophage cells. HPAC reduced LPS-induced inflammatory mediators such as IL-6, IL-1*β*, TNF-*α*, NO, and PGE_2_ in RAW 264.7 cells. Interestingly, 15-PGDH appears to play a pivotal role rather than COX-2 and mPGES-1 when HPAC regulated PGE_2_ levels. Meanwhile, HPAC showed anti-inflammatory effects by blocking I*κ*B*α* phosphorylation and NF-*κ*B nuclear translocalization. Also, we found that HO-1 upregulation was mediated by the mitogen-activated protein kinase (MAPK) pathways in HPAC-treated RAW 264.7 cells. And, in RAW 264.7 cells challenged with LPS, HPAC restored HO-1 expression, leading to NF-*κ*B inhibition. Through further experiments using specific MAPK inhibitors, we found that, in response to LPS, the phosphorylated I*κ*B*α* and activated NF-*κ*B were attenuated by p38 MAPK/HO-1 pathway. Therefore, HPAC targeting both the I*κ*B*α*/NF-*κ*B complex and 15-PGDH may be considered as a potential novel anti-inflammatory agent derived from a natural source.

## 1. Introduction

Inflammation is a defensive mechanism which acts by removing harmful stimuli such as pathogens, damaged cells, and toxic compounds. It consists of several processes mediated by activated inflammatory and immune cells, including macrophages and monocytes, and incorporates a complex series of reactions regulated by cytokines, growth factors, nitric oxide (NO), and prostaglandins (PGs) produced by active macrophages [1, 2]. However, uncontrolled acute inflammation may become chronic, contributing to numerous chronic inflammatory diseases, including arthritis, autoimmune diseases, atherosclerosis, and chronic hepatitis [3, 4]. There is growing evidence that plant foods rich in dietary nutrients are beneficial by inhibiting inflammatory mediators and treating diseases related to such factors [5, 6]. Omega-3 fatty acid which is found in several types of nuts and seeds has been reported to protect against autoimmune diseases and atherosclerosis [7]. Curcumin found in *Curcuma longa* has shown potential effects on diseases such as rheumatoid arthritis, inflammatory bowel disease, and pancreatitis [8]. Ginger contains many phenolic compounds, such as gingerols and shogaols, which have been effective in reducing pain in patients with knee osteoarthritis [9]. Studies have described plant-derived dietary flavonoids, such as quercetin and kaempferol, found in different fruits and vegetables exert anti-inflammatory effects by regulating signaling pathways associated with inflammation [10, 11].

*Artemisia capillaris* Thunberg (AC), which belongs to the genus *Artemisia* and family Asteraceae, is a traditional herbal medicine widely used in East Asian countries to improve conditions such as pyrexia, pain, hepatotoxicity, inflammation, cholestasis, and jaundice [12–15]. Many studies have shown various biological activities of AC, such as hypoglycemic, hypolipidemic, anti-inflammatory, and anticarcinogenic activity [16–19]. AC contains several flavonoids, phenolic acids, terpenoids, chromones, and coumarins [20]. Constituents of AC, such as chlorogenic acid, ascorbic acid, scoparone, and essential oil, elicit potent anti-inflammatory and antioxidants effects [21–24]. In traditional herbal medicine, heat processing is a pharmaceutic method not only to enhance pharmacological effects but also to improve the therapeutic value by reducing toxic effects [25–27]. Heat processing increased the anti-inflammatory and antiproliferative effects of *Cuscuta campestris* seeds compared to unprocessing [28]. Comparing different fractions of raw and heat-processed *Xanthium sibiricum* Patr., heat processing reduced the cytotoxicity in MIHA liver cells [29]. Also, compared to the raw plant, the total gypenosides extracted from heat-processed *Gynostemma pentaphyllum* (Thunb.) Makino showed a higher antitumor effect against A549 lung cancer cells [30].

Previous studies have shown that extracts of AC, *Artemisia apiacea* Hance, and *Artemisia iwayomogi* inhibit lipopolysaccharide- (LPS-) induced inflammatory factors by suppressing NF-*κ*B activation in human HepG2 cells and mouse RAW264.7 cells [31–34]. However, reports on the effect or mechanism of heat-processed *Artemisia capillaris* Thunberg (HPAC) are scarce. The purpose of this study is to evaluate, for the first time, the anti-inflammatory effect of HPAC in LPS-treated mouse macrophage cells and determine the underlying molecular mechanism.

## 2. Materials and Methods

### 2.1. Materials and Chemicals

Dulbecco's modified Eagle's medium (DMEM), penicillin-streptomycin, and fetal bovine serum (FBS) from Thermo Fisher (Carlsbad, CA, USA); dimethyl sulfoxide (DMSO), lipopolysaccharide (LPS), 3-(4,5-dimethylthiazol-2-yl)-2,5 diphenyltetrazoliumbromide (MTT), and dexamethasone (DXM) from Sigma-Aldrich (Saint Louis, MO, USA); SB203580 (p38 inhibitor) and PD98059 (ERK inhibitor) from Tokyo Chemical Industry (TCI, Tokyo, Japan) and SP600125 (JNK inhibitor) from Sigma-Aldrich (Saint Louis, MO, USA); antibody against NF-*κ*B p65 from Abcam (Burlingame, CA, USA); specific antibodies used against iNOS, COX-2, HO-1, ERK, JNK, p38, I*κ*B*α*, p-ERK, p-JNK, p-p38, p-I*κ*B*α*, and *β*-actin from Cell Signaling Technology (Beverly, MA, USA); antibodies against mPGES-1 and 15-PGDH from Cayman Chemicals (San Diego, CA, USA) were used.

### 2.2. Preparation of Heat-Processed *Artemisia capillaris* Thunberg Extract

Dried AC was purchased from Sunilcrudedrugs (Hongcheon, Korea). The procedure to prepare HPAC follows a previous report with modification [35]. AC was soaked in 30% EtOH for 30 min. AC was roasted in a convection oven (JSOF-150, JS Research Inc., Korea) for 1 h 20 min in 200°C. To prevent the herb from burning, the herb was turned over every 5 min while roasting. HPAC with 2 L of 30% ETOH was boiled for 2 h in 100°C, following filtration and evaporation. The lyophilized HPAC was successively extracted with a yield (w/w) of 8.22%. The voucher specimen (HPAC: BON190527.145) was stored at the herbarium of Korean Medicine at Semyung University. The lyophilized powders were solubilized and diluted with DMSO before treatment.

### 2.3. Gas Chromatography-Mass Spectrometry (GC-MS) Analysis

The constituents of the HPAC extract were evaluated using GC-MS analysis. GC-MS analysis was performed on an Agilent 6890N GC system interfaced with a Leco Pegasus IV Time of flight Mass Spectrometer. The electron energy was −70 eV, and the ion source temperature was 220°C. Each sample (1 *μ*L, dissolved in MeOH) was injected in split mode (10 : 1) at 280°C and separated through a capillary column of DB-5MS (30 × 0.25 × 0.25) (Agilent J&W column). The initial oven temperature was 30°C, which was increased to 300°C at 10°C/min. Carrier gas (Helium) flow was 0.8 mL/min.

### 2.4. Cell Culture

RAW 264.7 cells were obtained from Korean Cell Line Bank (KCLB) and incubated in high-glucose DMEM supplemented with 10% fetal bovine serum (FBS), 100 IU/mL penicillin, and 100 *μ*g/mL streptomycin under an atmosphere containing 5% CO_2_ at 37°C in an incubator.

### 2.5. MTT Cytotoxicity Assay

The cytotoxicity of HPAC on RAW 264.7 cells was assessed via colorimetric MTT assay. RAW 264.7 cells were grown at a density of 1 × 10^4^ cells per well in 96-well plate. After 24 h, cells were then treated with different concentrations of HPAC and incubate for 24 hr. Following treatment, 20 *μ*L of MTT (5 mg/mL) solution was added to each well and further incubated for 4 h at 37°C. Next, the media (containing MTT solution) were removed from each well and 100 *μ*L DMSO was added to each well to dissolve the resulting formazan crystals. Finally, the plate was smoothly agitated for 10 min on a shaker, and the absorbance was then measured at 570 nm using a microplate reader (BioTek, Winooski, VT, USA). The results were shown as relative cell viability referred to as control (equal to 100%).

### 2.6. Measurement of Nitric Oxide (NO) Production

NO concentration in the cell culture medium was determined using a colorimetric assay based on the Griess reaction. To measure NO secretion, RAW 264.7 cells were seeded in 96 well plates, 5 × 10^4^ cells per well. After 24 h, cells were pretreated with different concentrations of HPAC for 2 h and then stimulated with 1 mg/mL of LPS for an additional 24 h. After LPS stimulation, a cell culture medium was collected to measure the amount of NO using the Griess reagent. In brief, 50 *μ*L of Griess reagent was added to an equal volume of cell culture medium in a 96-well plate and the plate was smoothly agitated for 10 min on a shaker at room temperature. Finally, the concentration of nitrite was calculated from a standard curve of known concentrations of sodium nitrite dissolved in DMEM.

### 2.7. Determination of IL-6, IL-1*β*, TNF-*α*, and PGE2

RAW 264.7 cells were plated in a 24-well plate at a density of 1 × 10^5^ cells/well and incubated overnight. The cells were pretreated with different concentrations of HPAC for 2 h and then challenged with LPS for another 24 h. The supernatants were then centrifuged at 12000 rpm at 4°C for 5 min to discard cell debris and the remnant media was collected. The levels of IL-6, TNF-*α,* and IL-1*β* were measured by enzyme-linked immunosorbent assay using ELISA kit (Invitrogen, Carlsbad, CA, USA) and PGE_2_ were analyzed using ELISA kit (R&D Systems, Minneapolis, MN, USA) according to the manufacturer's instructions.

### 2.8. Extraction of Nuclear and Cytosolic Fraction

Cytoplasmic and nuclear extracts were prepared using a Nuclear and Cytoplasmic Extraction Reagents kit (Thermo Fisher Scientific, Rockford, IL, USA) following the manufacturer's instructions. Briefly, the RAW 264.7 cells were seeded in 6-well plates at a density of 5 × 10^5^ cells/well and incubated for 24 h. Next, the cells were pretreated with different concentrations of HPAC for 2 h and then stimulated with LPS for another 2 h. The cells were then harvested with PBS and centrifuged. Next, an ice-cold cytoplasmic protein extraction solution was added and centrifuged to separate the cytoplasmic extract. Then, the cytoplasmic extract was transferred to clean prechilled tubes and the pellets produced were prepared for nuclear extraction by adding ice-cold nuclear protein extraction solution.

### 2.9. Western Blotting

Cells were lysed in RIPA lysis buffer (25 mM Tris HCl pH 7.6, 150 mM NaCl, 1% NP-40, 1% sodium deoxycholate, 0.1% sodium dodecyl sulfate (SDS)) with protease inhibitor cocktail (Roche, Mannheim, Germany). The protein concentration was determined using the bicinchoninic acid protein assay kit (Thermo Scientific, Rockford, IL, USA). Total protein lysates were separated by SDS-PAGE and transferred onto nitrocellulose membranes. The membranes were then blocked for 1 h at room temperature in 5% skim milk, followed by an overnight incubation at 4°C with a specific primary antibody. The next day, the membranes were washed and incubated for an additional 1 h with HRP-conjugated secondary antibody (1 : 5000) at room temperature after thoroughly washing three times with TBST. Bands were detected by ECL (LPS Solution, Daejeon, Korea), and band intensities were quantified using ImageJ gel analysis software.

### 2.10. Statistical Analysis

Statistical calculations were done in GraphPad Prism version 5. Results are presented as means ± SEM. Data were analyzed by one-way ANOVA followed by post hoc Tukey's test. *p* < 0.05 indicates statistical significance.

## 3. Results

### 3.1. Gas Chromatography-Mass Spectrometry (GC-MS) Analysis

**Figure 1:**
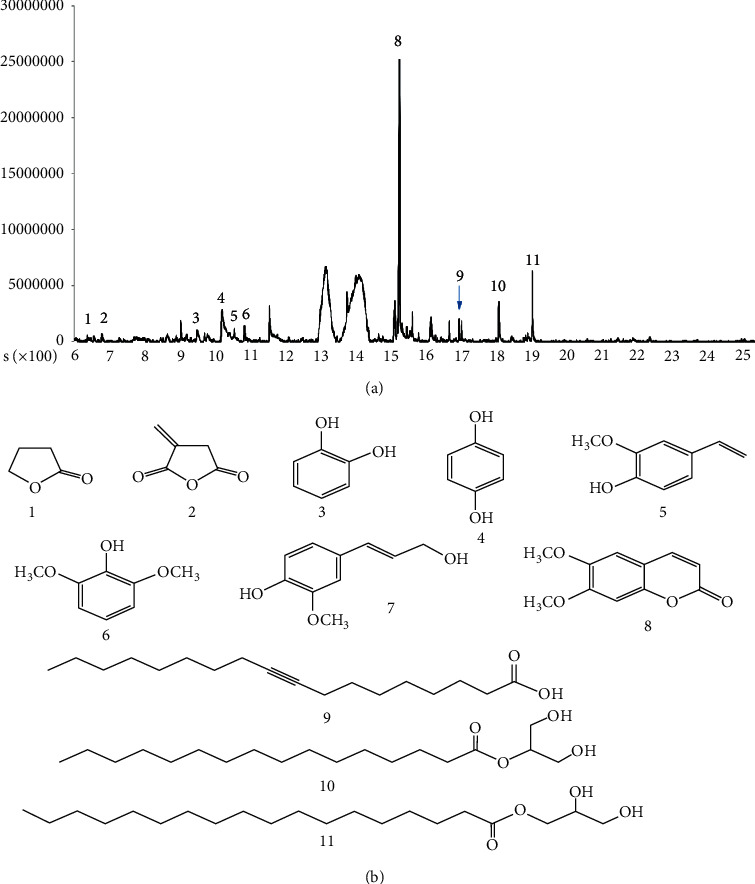
GC-MS analysis of HPAC. (a) GC-MS chromatograms of HPAC. (b) Structure of candidate compounds by GC-MS. The HPAC extract contains lactones (1, 2), phenolics (3–8), fatty acids (9), and fatty acids ester (10, 11).

HPAC extract was analyzed using a GC-MS and compared with standards from the library database. Butyrolactone (1), dihydro-3-methylene-2,5-furandione (2), catechol (3), hydroquinone (4), 2-methoxy-4-vinylphenol (5), 2,6-dimethoxy-phenol (6), scoparone (8), 9-octadecynoic acid (9), 2-hydroxy-1-(hydroxymethyl)ethyl ester hexadecanoic acid (10), and 2,3-dihydroxypropyl ester octadecanoic acid (11) were observed in HPAC extract (Figure 1).

### 3.2. Cell Viability and Cytokine Production

**Figure 2:**
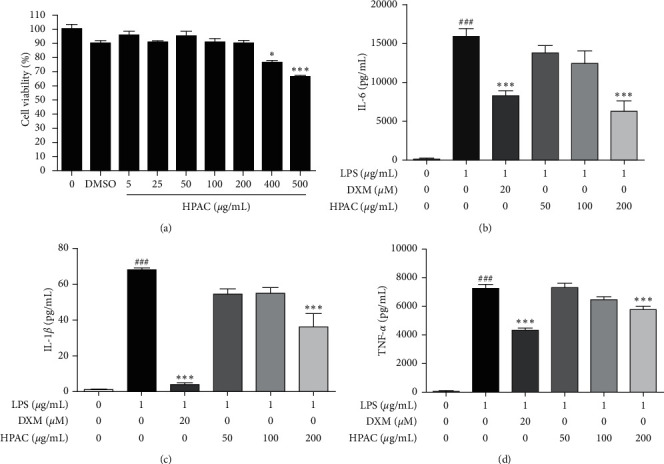
Effects of HPAC on cell viability and cytokine production. (a) RAW 264.7 cells were treated with 5–500 *μ*g/mL of HPAC and cell viability was determined by MTT assay. RAW 264.7 cells pretreated with indicated concentration of HPAC followed by LPS for 24 h and the production of (b) IL-6, (c) IL-1*β,* and (d) TNF-*α* were determined by ELISA. DXM was used as a positive control. The data are presented as means ± SEM (*n* = 3). (a) ^*∗∗∗*^*p* < 0.001 versus DMSO group, (b– d) ^###^*p* < 0.001 versus control group, ^*∗∗∗*^*p* < 0.001 versus LPS group.

RAW 264.7 cells were treated with different concentrations of HPAC (5 *μ*g/mL to 500 *μ*g/mL) and cell viability was performed by MTT assay. After HPAC treatment, significant cell toxicity was observed at 400 *μ*g/mL, and lower concentrations (5 *μ*g/mL to 200 *μ*g/mL) did not show significant changes (Figure 2(a)). Therefore, the concentrations of HPAC ranging from 50 *μ*g/mL to 200 *μ*g/mL were employed for further study. Dexamethasone (DXM), a classic steroidal anti-inflammatory and immunomodulatory drug, was included as the positive control in the study [36]. TNF-*α*, IL- 6, and IL-*β* cytokines play essential roles in inflammatory responses; thus, we investigated whether HPAC could inhibit secretion of these cytokines in macrophages stimulated by LPS. After LPS activation, the results show that IL-6 (147-fold), IL-*β* (68-fold), and TNF-a (93-fold) levels significantly increased compared to control (Figures 2(b)–2(d)). HPAC treatment suppressed the production of these cytokines in a dose-dependent manner; however, significance was achieved only at 200 ug/mL (Figures 2(b)–2(d)). The levels of IL-6 (0.4-fold), IL-*β* (0.5-fold), and TNF-a (0.8-fold) changed significantly in the HPAC-treated group (200 ug/mL) compared to the LPS-treated group. After LPS stimulation, the HPAC-treated group demonstrated a lesser effect on inhibiting TNF-*α* and IL-*β* production compared to the DXM-treated group (Figures 2(c) and 2(d)). However, HPAC treatment showed stronger IL-6 inhibition in comparison to DXM at 200 *μ*g/mL (Figure 2(b)). These results indicate that HPAC inhibits the LPS-induced release of proinflammatory cytokines in RAW 264.7 cells.

### 3.3. HPAC Regulates NF-*κ*B Nuclear Translocation

**Figure 3:**
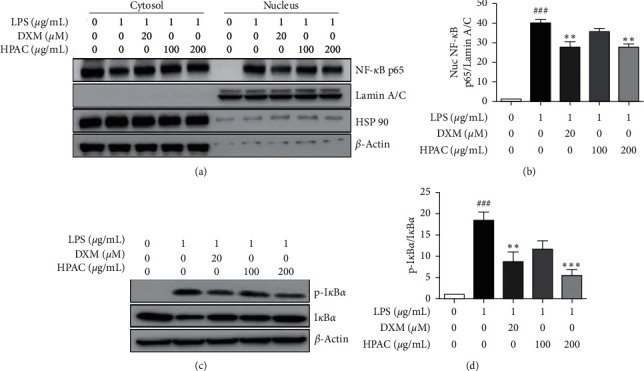
Effect of HPAC on NF-*κ*B nuclear translocation. RAW 264.7 cells were pretreated with the indicated concentrations of HPAC for 2 h followed by LPS for 2 h. (a) Nuclear and cytosolic proteins were isolated and analyzed by western blotting using anti-NF-*κ*B p65 antibody. Lamin A/C, HSP 90, and *β*-actin were used as internal controls for the nuclear and cytosolic fractions, respectively. (b) Quantitative analysis of nuclear NF-*κ*B p65 expression relative to Lamin A/C. (c) Western blot analysis of p-I*κ*B*α*, I*κ*B*α*, and *β*-actin. (d) Quantitative analysis of p-I*κ*B*α* expression relative to I*κ*B*α*. DXM was used as a positive control. The data are presented as means ± SEM (*n* = 3). ^###^*p* < 0.001 versus control group, ^*∗∗*^*p* < 0.01 and ^*∗∗∗*^*p* < 0.001 versus LPS group.

NF-*κ*B has been reported to be a positive regulator of numerous inflammatory mediators and proinflammatory cytokines [37, 38]. So we hypothesized that the inhibitory effect of HPAC on cytokine secretion may be likely via NF-*κ*B regulation. To elucidate the inhibitory effect of HPAC on NF-*κ*B nuclear expression, RAW 264.7 cells were pretreated with the indicated concentrations (100 and 200 *μ*g/mL) of HPAC for 2 h and then stimulated with LPS for 2 h. Results showed that the protein levels of NF-*κ*B p65 in the nuclear fraction significantly increased in the LPS-treated group (40-fold), whereas HPAC treatment inhibited the nuclear translocation of NF-*κ*B p65 (Figures 3(a) and 3(b)). The nuclear expression of NF-*κ*B p65 in HPAC-treated cells (200 *μ*g/mL) showed a 0.7-fold change compared to LPS-treated cells (Figure 3(b)).

NF-*κ*B transcriptional activity is suppressed by a stable I*κ*B/NF-*κ*B complex whereas activated I*κ*B kinase, by inflammatory stimulus, phosphorylates I*κ*B leading to ubiquitination and its degradation [39]. To explore how HPAC prevents LPS-induced NF-*κ*B activation, we investigated the inhibitory effect of HPAC on the phosphorylation of I*κ*B*α*, which led to NF-*κ*B activation. Our data show that LPS strongly phosphorylated I*κ*B*α*, by a 19-fold change, compared to control. And HPAC treatment attenuates the increased phosphorylation of I*κ*B*α*, by 0.8- and 0.3-fold, compared to LPS treatment (Figures 3(c) and 3(d)). These results suggest that HPAC reduced NF-*κ*B nuclear translocation by inhibiting I*κ*B*α* phosphorylation.

### 3.4. HPAC Regulates NO and PGE2 Production

**Figure 4:**
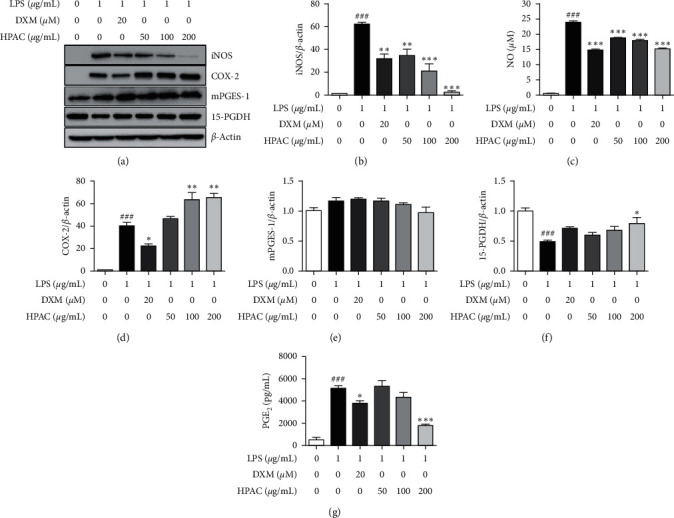
Effect of HPAC on NO and PGE_2_ production. RAW 264.7 cells treated with indicated concentrations of HPAC for 2 h followed by LPS for 24 h. (a) Western blot analysis of iNOS, COX-2, mPGES-1, 15-PGDH, and *β*-actin. Quantitative analysis of (b) iNOS, (d) COX-2, (e) mPGES-1, and (f) 15-PGDH expression relative to *β*-actin. (c) NO and (g) PGE_2_ production were determined by Griess assay and ELISA, respectively. DXM was used as a positive control. The data are presented as means ± SEM (*n* = 3). ^###^*p* < 0.001 versus control group, ^*∗*^*p* < 0.05, ^*∗∗*^*p* < 0.01, and ^*∗∗∗*^*p* < 0.001 versus LPS group.

NO is a vital proinflammatory mediator, and excessive NO production is involved in the pathogenesis of many inflammatory diseases [40, 41]. Activation of iNOS, mainly regulated by NF-*κ*B, increases NO production [42, 43]. Therefore, we examined the inhibitory effect of HPAC on LPS-induced iNOS and NO levels in RAW 264.7 cells. As shown in Figures 4(a) and 4(b), iNOS expression was remarkably elevated by LPS stimulation and HPAC significantly attenuated the protein expression of iNOS in a dose-dependent manner. Furthermore, HPAC achieved the strongest inhibition (0.04-fold) at 200 *μ*g/mL, which was more significant than DXM (0.5-fold) (Figure 4(b)). Next, NO production was analyzed by measuring the accumulation of nitrites in the supernatants using Griess assay. HPAC treatment significantly inhibited LPS-induced NO production dose-dependently (Figure 4(c)).

PGE_2_ has been implicated in various biological actions, such as pain sensation and inflammatory condition [44]. COX-2 and mPGES-1 are functionally coupled and considered as the primary enzymes for the inflammatory PGE_2_ generation [45]. Although the positive control DXM showed significant inhibitory effects against COX-2 expression, HPAC had no effect in decreasing COX-2 and mPGES-1 expression (Figures 4(a), 4(d), and 4(e)). 15-PGDH is identified as a catabolizing enzyme that converts PGE_2_ into its inactive product [45]. HPAC treatment showed a dose-dependent increase in 15-PGDH expression and the maximum effect was achieved at 200 *μ*g/mL (1.6-fold) (Figures 4(a) and 4(f)). Taken together, these results indicate that the inhibitory effect of HPAC on PGE_2_ production is mediated by 15-PGDH upregulation (Figure 4(g)).

### 3.5. HPAC Regulates HO-1 via MAPK Signaling

**Figure 5:**
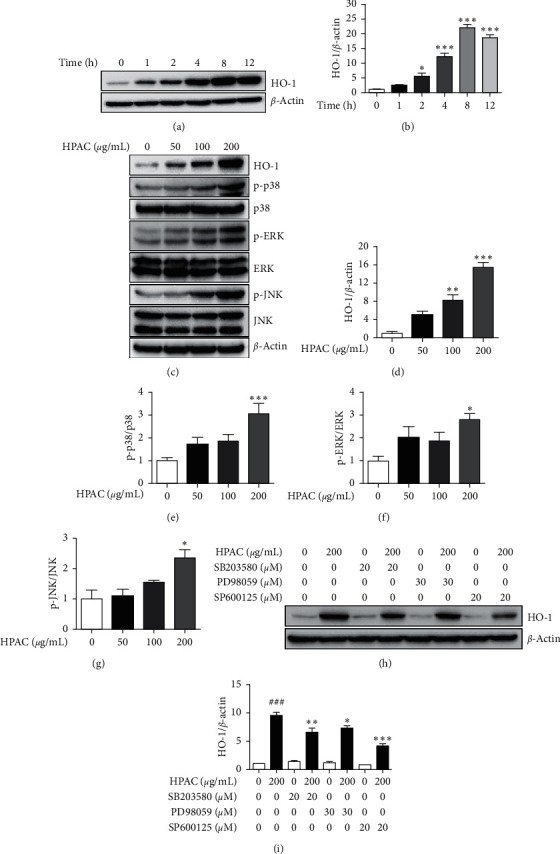
Effects of HPAC on HO-1 via MAPK signaling. RAW 264.7 cells were treated with HPAC 200 *μ*g/mL for indicated times. (a) Western blot analysis of HO-1 and *β*-actin. (b) Quantitative analysis of HO-1 expression relative to *β*-actin. RAW 264.7 cells were treated with indicated concentration of HPAC for 4 h. (c) Western blot analysis of HO-1, p-p38, p38, p-ERK, ERK, p-JNK, JNK, and *β*-actin. Quantitative analysis of (d) HO-1 expression relative to *β*-actin, (e) p-p38 expression relative to p38, (f) p-ERK expression relative to ERK, and (g) p-JNK expression relative to JNK. RAW 264.7 cells were pretreated with specific MAPK inhibitors, SB203580 (20 *μ*M), PD98059 (30 *μ*M), and SP600125 (20 *μ*M), for 1 h and then HPAC 200 *μ*g/mL for 4 h. (h) Western blot analysis of HO-1 and *β*-actin. (i) Quantitative analysis of HO-1 expression relative to *β*-actin. The data are presented as means ± SEM (*n* = 3). ^###^*p* < 0.001 versus control group, ^*∗*^*p* < 0.05, ^*∗∗*^*p* < 0.01, and ^*∗∗∗*^*p* < 0.001 versus HPAC group.

HO-1 is an important component of the cellular defense against inflammation [46], we further examined whether HPAC could induce HO-1 expression. Our data show that by treating RAW 264.7 cells at a fixed concentration of 200 *μ*g/mL, HO-1 was expressed as early as 2 h and continuously increased until 8 h, after which the expression was reduced (Figures 5(a) and 5(b)). Also, in accordance with the different concentrations of HPAC, HO-1 expression significantly increased dose-dependently (Figures 5(c) and 5(d)). Next, we investigated the underlying mechanism of HO-1 induction by HPAC treatment. MAPK family signaling cascades have been reported to induce HO-1 expression [47–49]. Therefore, in the current study, the effects of HPAC on phosphorylation levels of MAPKs including JNK, p38, and ERK were analyzed. While phosphorylation of p38, ERK, and JNK MAPKs increased by HPAC treatment, significance only occurred at the highest concentration (Figures 5(e)–5(g)). The significant upregulation of p38, ERK, and JNK at 200 *μ*g/mL was in parallel with HO-1 induction (Figure 5(d)). In addition, HPAC treatment activated p38 (3.8-fold) to a greater extent than ERK (2.8-fold) and JNK (2.4-fold) (Figures 5(e)–5(g)). To further verify the involvement of MAPK pathway in the upregulation of HO-1 expression by HPAC, the effects of MAPK specific inhibitors, p38 (SB203580), ERK1/2 (PD98059), and JNK (SP600125), were analyzed. As indicated in Figures 5(h) and 5(i), treating the cells with HPAC significantly increased the level of HO-1 by 10-fold, whereas SB203580, PD98059, and SP600125 administration attenuated HO-1 expression by 0.7-, 0.8-, and 0.4-fold, respectively. Taken together, these results indicate that the upregulation of HO-1 expression by HPAC was mediated via the MAPK pathway in RAW 264.7 cells.

### 3.6. HPAC Regulates I*κ*B*α*/NF-kB Pathway by HO-1 Activation

**Figure 6:**
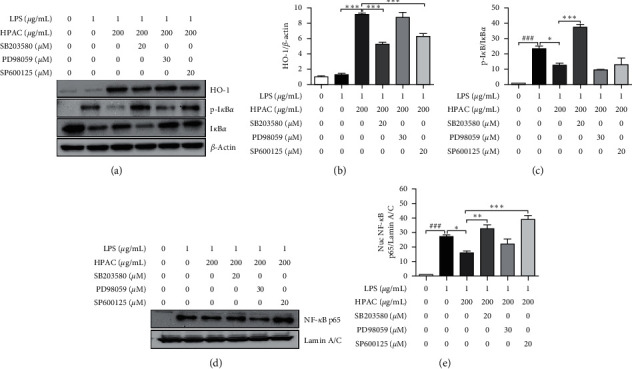
Effects of HPAC on IkB*α*/NF-kB pathway by HO-1 activation. RAW264.7 cells were pretreated with MAPK specific inhibitors, SB203580 (20 *μ*M), PD98059 (30 *μ*M), and SP600125 (20 *μ*M), for 1 h and then HPAC 200 ug/mL for 2 h followed by LPS for 2 h. (a) Western blot analysis of HO-1, p-I*κ*B*α*, I*κ*B*α*, and *β*-actin. Quantitative analysis of (b) p-I*κ*B*α* expression relative to I*κ*B*α*; (c) HO-1 expression relative to *β*-actin. (d) Nuclear and cytosolic extracts were isolated and levels of NF-*κ*B p65 in nuclear fraction were determined using western blot analysis. (e) Quantitative analysis of NF-*κ*B p65 expression relative to Lamin A\C. The data are presented as means ± SEM (*n* = 3). ^####^*p* < 0.001 versus control group, ^*∗*^*p* < 0.05, ^*∗∗*^*p* < 0.01, and ^*∗∗∗*^*p* < 0.001 versus HPAC group.

Our results found that HPAC upregulates HO-1 expression via activating MAPK. Furthermore, HPAC prevented NF-*κ*B p65 nuclear translocation by suppressing I*κ*B*α* phosphorylation. Previously, it has been reported that HO-1 inhibits NF-*κ*B nuclear translocation [50, 51]. To clarify, whether HPAC inhibits NF-*κ*B p65 nuclear translocation via inducing HO-1 expression through activating MAPK, the effect of MAPK inhibitors on HPAC-induced HO-1 expression and I*κ*B*α* phosphorylation was evaluated in LPS-challenged cells. Despite the fact that SB203580, PD98059, and SP600125 reversed the HPAC-induced HO-1 expression (Figures 5(h) and 5(i)), interestingly, SB203580 and SP600125 significantly reversed the HPAC-mediated HO-1 upregulation in LPS-treated condition. However, PD98059 had no effect on HO-1 expression in the LPS-treated condition (Figures 6(a)–6(c)). Furthermore, only SB203580 (but not PD98059 and SP600125) markedly enhanced the repressed I*κ*B*α* phosphorylation by HPAC treatment in the LPS-treated condition. As shown in Figures 3(a) and 3(b), HPAC prevented NF-*κ*B p65 nuclear translocation. In contrast, the nuclear accumulation of NF-*κ*B p65 was rescued by SB203580 and SP600125 (Figures 6(d) and 6(e)). Together, these results suggest that p38 MAPK-dependent HO-1 may play a functional role in regulating NF-*κ*B by HPAC under inflammatory conditions.

## 4. Discussion

Processing, also known as *Paozhi* in Chinese or *Poje* in Korean, is a traditional pharmaceutic method involving techniques such as stir-frying, stewing, boiling, and steaming [52]. Before clinical application, different processing techniques are used to reduce and prevent toxicity and to induce effectiveness via guidance and concentration [53]. Studies show that processing reduced cytotoxicity of *Gardenia jasminoides* Ellis. and *Xanthium sibiricum* Patr. extracts compared to raw extracts [29, 54]. Both HPAC and AC treatment showed significant cytotoxicity at 400 and 500 *μ*g/mL, but HPAC was considerably less toxic (Supplementary Figure 1).

Inflammation is a complex biological process regulated by inflammatory mediators such as TNF-*α*, IL-1*β*, IL-6, NO, and PGE_2_. The excessive production of proinflammatory cytokines is generally recognized to play key roles in the development of inflammatory diseases [55, 56]. DXM is a potent synthetic corticosteroid that exhibits anti-inflammatory and immunosuppressive effects [36]. DXM is widely used for the treatment of pneumonia, bronchiolitis, and rheumatoid arthritis [57–59]. The DXM-treated group was considered as the positive control. Numerous studies have shown that crude extracts and compounds isolated from AC possess anti-inflammatory effects in different cell types [21, 22, 34, 60]. In parallel, our data demonstrate that HPAC reduces inflammatory responses in LPS-activated macrophage cells. The secretion of proinflammatory cytokines, including TNF-*α*, IL-6, and IL-1*β*, were inhibited by HPAC treatment (Figures 2(b)–2(d)). In order to identify the potential mechanism of action, we analyzed the NF-*κ*B pathway. NF-*κ*B is a well-characterized transcriptional regulator responsible for promoting the production of inflammatory mediators [37, 38]. The activation of NF-*κ*B is accompanied by I*κ*B phosphorylation and degradation, leading to NF-*κ*B nuclear translocation [61, 62]. HPAC significantly attenuated the nuclear translocation of NF-*κ*B p65 through coordinated regulation of I*κ*B*α* phosphorylation (Figures 3(a)–3(d)), which perhaps explains the decrease in IL-6, IL-1*β*, and TNF-*α* production (Figures 2(b)–2(d)).

In addition, NF-*κ*B is a pivotal transcription factor that regulates the expression of iNOS and COX-2 enzymes [42]. The excessive production of iNOS-derived NO can act as modulators of inflammation [42]. HPAC significantly diminished the iNOS and NO levels in LPS stimulated RAW 264.7 cells (Figures 4(a)–4(c)). However, the HPAC-mediated downregulation of NF-*κ*B did not correlate with COX-2-dependent PGE_2_ synthesis (Figures 4(a)–4(g)). Earlier reports demonstrated that AC and its constituents inhibit the expression of COX-2 and PGE_2_ in cooperation with NF-*κ*B suppression [31, 63, 64]. In light of this controversial observation, we investigated other enzymes regulating the synthesis (mPGES-1) and catabolism (15-PGDH) of PGE_2_. HPAC showed no effect on mPGES-1 expression (Figure 4(e)). Interestingly, HPAC ameliorated the LPS-induced decrease of 15-PGDH (Figure 4(f)). 15-PGDH rapidly oxidizes 15-(S)-hydroxyl group of PGE_2_ and leads to its degradation [45]. Our data suggest that HPAC potentiates reduction of proinflammatory lipid mediator PGE_2_ by 15-PGDH-dependent and COX-2/mPGES-1-independent manner. Several studies have reported agents that regulate the tumor-suppressing role of 15-PGDH. Cancer cell lines treated with indomethacin, TGF-1*β*, calcitriol, and histone deacetylase inhibitors activated the 15-PGDH transcription rates and induced antiproliferative effects [65–68]. One report demonstrates that 15-PGDH is activated by IL-4 in association with JAK/STAT6, MAPK, PI3K, and PKC signaling in lung cancer cells [69]. Recent reports suggest that 15-PGDH is modulated by microRNAs (miRNA). In many types of tumors, miRNA-21 binds to the 3′-UTR site of 15-PGDH mRNA and inhibits 15-PGDH expression, which in turn promotes tumor growth [70–73]. In cervical cancer, miR-146b-3p negatively regulated 15-PGDH involving STAT3 and AKT signaling [74]. One study demonstrates that tumor radioresistance is mediated by miR-620 targeting 15-PGDH [75]. Further research is needed to fully understand how HPAC regulates 15-PGDH.

HO-1 has been reported to negatively regulate NF-*κ*B and inhibit the production of proinflammatory mediators [50, 51]. Multiple mechanisms have been reported to induce the expression of HO-1, including MAPK pathway [47–49]. Since HPAC elevated HO-1 expression in a time- and dose-dependent manner, the association between HO-1 and MAPK pathway was examined. HPAC induced the phosphorylation of p38, ERK, and JNK in parallel with HO-1 expression (Figures 5(c)–5(g)). Moreover, we also determined the effect of specific MAPK inhibitors on HPAC-induced HO-1. Our experiment shows that all three inhibitors (SB203580, PD98059, and SP600125) significantly reversed the HO-1 increase by HPAC. Our data confirm that MAPK signaling is involved in HPAC-mediated regulation of HO-1 (Figures 5(h) and 5(i)). Based on the inhibitory effects of HPAC on LPS-induced NF-*κ*B activation, we asked whether HO-1 was linked to these effects. Our results show that HO-1 was effectively increased, whereas phosphorylated I*κ*B*α* was markedly decreased by HPAC in LPS-treated cells (Figures 6(a)–6(c)). Previous studies have shown that blocking HO-1 restored I*κ*B*α* phosphorylation and NF-*κ*B nuclear translocation, thereby interfering with the anti-inflammatory effect of chlorogenic acid and lycopene [76, 77]. To further verify the effect of HO-1 on the crosstalk between MAPK pathway and NF-*κ*B pathway, we blocked the HO-1 activity using specific MAPK inhibitors. Despite the fact that all three inhibitors blocked HO-1 activity in HPAC-treated RAW 264.7 cells, the MAPK inhibitors vary in their effect to reverse the expression of HO-1 induced by HPAC in LPS-challenged cells. Both SB203580 and SP600125 achieved HO-1 downregulation and nuclear NF-*κ*B p65 upregulation; however, PD98059 showed no effect (Figures 6(a)–6(e)). Although HO-1 was suppressed, SP600125 was not sufficient to induce phosphorylation-induced degradation of I*κ*B*α* compared to SB203580 (Figures 6(a)–6(c)). Interestingly, one study suggests crosstalk between JNK and NF-*κ*B pathway in TNF-*α*-stimulated HepG2 cells [78]. The JNK inhibitor SP600125 reduced HSP27 phosphorylation, which plays a crucial role in the binding ability of IKK with I*κ*B*α* [78]. It may be possible that SP600125 interfered with I*κ*B*α* phosphorylation. Even though I*κ*B*α* phosphorylation and degradation are impaired by JNK inhibition, the increased NF-*κ*B nuclear translocation remains unclear. Nonetheless, only the p38/HO-1 signaling pathway seems to be required for phosphorylation and degradation of I*κ*B*α*. Our results indicate the importance of p38/HO-1 signaling pathway for HPAC-mediated regulation on I*κ*B*α*/NF-*κ*B complex under LPS inflammatory condition.

## 5. Conclusions

AC has been widely consumed as a dietary product in Asia. Since heat processing is used to promote drug effectiveness and safety in herbal medicine, we provide evidence that HPAC decreases inflammatory responses. Previous studies have shown that pure capillarisin (CAP) isolated from AC showed muscle protective effect through MAPK and NF-*κ*B signaling [79]. HPAC exhibits similar effects but its anti-inflammatory effect is through regulating I*κ*B*α* phosphorylation and NF-*κ*B activation via HO-1 induction. Moreover, p38 MAPK signaling is required for HO-1 activation by HPAC in LPS-treated RAW 264.7 cells. Also, it is known that aerial parts of AC and some of its coumarin and flavonoid derivatives show anti-inflammatory activity via 5-lipoxygenase (LOX) inhibition [80]. Interestingly in this study, inhibition of PGE_2_ secretion was mediated through 15-PGDH upregulation and not through COX-2 inhibition. Regulatory effects of HPAC on I*κ*B*α*/NF-*κ*B complex and 15-PGDH may explain the anti-inflammatory activity and HPAC may serve as a candidate for developing anti-inflammatory agents.

There have been reports to achieve optimal extraction temperatures for enhancing HPAC anti-inflammatory capacity [81]. Further in vivo studies and experiments regarding heat processing conditions may help better understand the (drug) mechanism of HPAC and aid its development as an anti-inflammatory agent.

## Data Availability

The data used to support the findings of this study are available from the corresponding author upon request.

## Supplementary Materials

Supplementary Materials Supplementary Figure 1: the cytotoxicity of the AC on RAW 264.7 cells.
