# Supplementary material for: Anti-Inflammatory Effects of Heat-Processed Artemisia capillaris Thunberg by Regulating IκBα/NF-κB Complex and 15-PGDH in Mouse Macrophage Cells
Source: Evid Based Complement Alternat Med. 2021 Jun 7;2021:5320314. doi: 10.1155/2021/5320314 (PMC8203361; doi:10.1155/2021/5320314)
Supplement: Supplementary Materials — Supplementary Figure 1: the cytotoxicity of the AC on RAW 264.7 cells. [file 5320314.f1.docx]

**Supplementary material**


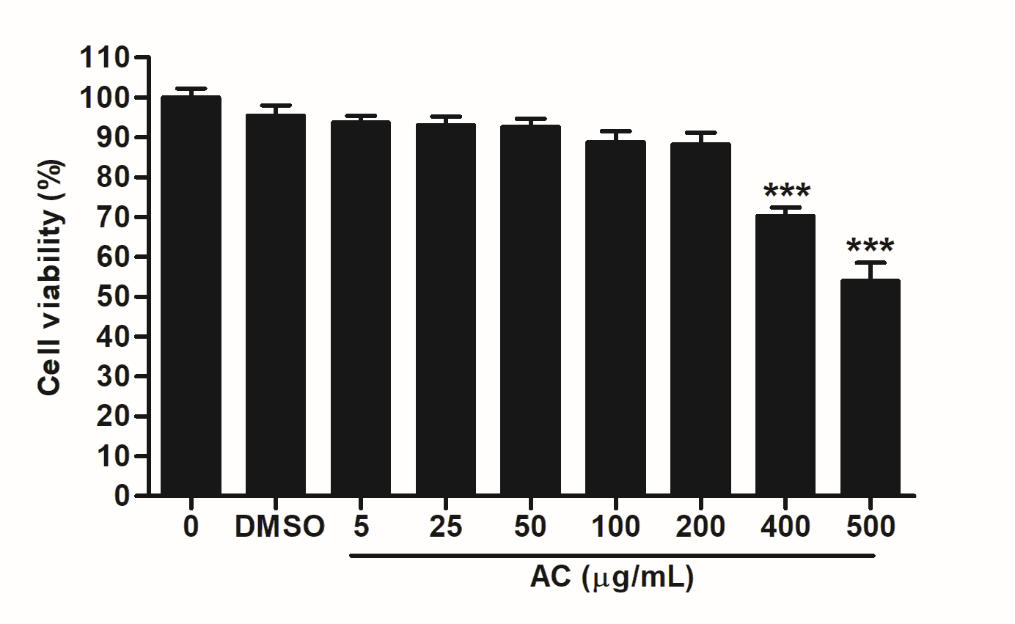


Supplementary Figure 1. Effects of AC on cell viability. RAW 264.7 cells were treated with 5-500 µg/mL of AC and cell viability was determined by MTT assay. The data are presented as means ± S.E.M. (n = 3). *** *p* < 0.001 vs DMSO group.
